# Raman Spectroscopy as Molybdenum and Tungsten Content Analysis Tool for Mesoporous Silica and Beta Zeolite Catalysts

**DOI:** 10.3390/molecules25214918

**Published:** 2020-10-23

**Authors:** Romana Velvarská, Zdeněk Tišler, Veronika Raichlová, José Miguel Hidalgo-Herrador

**Affiliations:** Unipetrol Centre for Research and Education, Revoluční 1521/84, 400 01 Ústí nad Labem, Czech Republic; zdenek.tisler@unicre.cz (Z.T.); veronika.raichlova@unicre.cz (V.R.); jose.hidalgo@unicre.cz (J.M.H.-H.)

**Keywords:** Raman spectroscopy, tungsten, molybdenum, quantitative analyses, calcination

## Abstract

Raman spectroscopy was used for the quantitative determination of Mo and W in Mo- and W-supported mesoporous silica (Mo/SBA-15 and W/SBA-15, respectively) and Mo-supported beta zeolite (Mo-BEA). Three Raman quantitative models were developed and optimized for the metal contents of Mo/SBA-15, W/SBA-15, and Mo/BEA. Subsequently, the models were characterized using the root mean square error of calibration (RMSEC), root mean square error of cross-validation (RMSECV), root mean square error of prediction (RMSEP), correlation coefficient, and predicted residual error sum of squares (PRESS) diagnostic function. The calibration range of the models were in the range of approximately 2–40 wt% for the SBA-15 support and 1–21 wt% for the BEA support because the BEA support presented lower Mo absorption than the SBA-15 support. The RMSEC, RMSECV, and RMSEP values were below 1.80% for all developed models. The highest and lowest correlation coefficients corresponded to the W/SBA-15 (0.9984) and Mo/BEA (0.9777) models, respectively. The change in catalyst support affected the mentioned chemometric parameters (Mo/SBA-15 vs. Mo/BEA). Subsequently, Raman spectroscopy combined with the temperature control stage was used to study the calcination of Mo/BEA, Mo/SBA-15, and W/SBA-15 using three-dimensional diagrams, in which the changes in catalyst structure were analyzed as functions of the temperature and time. Raman spectroscopy was determined to be a suitable analytical tool for the quantitative analysis of the metal contents of the catalyst and optimization of the calcination process. Therefore, Raman spectroscopy can be used during catalyst manufacturing.

## 1. Introduction

During the quality control step, which is the last step of production processes and includes quantitative and qualitative evaluation, it is determined whether the final product meets consumers’ demands. Quality control is typically performed using fast, accurate, and effective analytical methods [1].

The production and processing of chemical catalysts are not significantly different from other production processes, and therefore, quality control ultimately determines the applicability of the final products. The catalysts must meet a number of chemical and physical properties that are determined based on ASTM standards. Analytical methods such as potentiometric titration, wet chemistry, and especially methods for determining metal content are used to determine the chemical composition. However, the analytical methods used to control the metal contents of catalysts, such as X-ray fluorescence, inductively coupled plasma optical emission spectroscopy (ICP-OES), and atomic absorption spectroscopy, are time-consuming, expensive, and limited by parameters such as the state and amount of sample or the destructive nature of the analysis. These techniques are not universal, and it is often necessary to combine several methods, which increases production costs [2,3,4,5,6,7].

Raman spectroscopy is a potential fast quality control monitoring method. This method, which focuses on the analysis of the vibrational and rotational states of atoms and molecules, is complementary to infrared (IR) spectroscopy, but its advantages lie mainly in the possibility of selecting the excitation laser wavelengths to obtain a strong Raman signal of samples and avoiding of fluorescence. In contrast to IR spectroscopy, Raman spectroscopy analyzes scattering, and, consequently, molecules that cannot be observed using IR spectroscopy can be analyzed using Raman spectroscopy if they could change the polarizability of the light incident on their bonds (Raman active vibration). Optimized Raman spectra can be used for qualitative and quantitative analysis purposes. Suitable excitation lasers should be used for different substances because different wavelengths are required to observe different Raman vibrations, which can be used to study the structure of substances [8,9,10].

Raman spectroscopy is widely used for quality control in the pharmaceutical industry and its applications are on the rise in the chemical industry. In the 1980s, Boerio and Koenig reported the application and suitability of Raman spectroscopy for the study of polymeric materials [11]. Nowadays, Raman spectroscopy is used to study the polymerization of substances in aqueous media [12] or the kinetics of polymerization reactions [13]. However, Raman spectroscopy has not been validated yet as an analytical technique for the rapid screening of industrial catalysts in oxide form. Owing to its advantages, such as analysis speed, the possibility of using handheld analyzers without the need for laboratory equipment, and versatile applications, this analytical spectral technique was used to determine the Mo and W contents of Mo- and W-supported mesoporous silica (SBA-15) and Mo-supported beta zeolite (BEA) catalysts.

Molybdenum and tungsten oxides are commonly used in the refining and petrochemical industries, and their catalytic effects during biomass processing have been studied. Owing to their properties, Mo and W affect the yields and conversions of several chemical processes. Molybdenum oxides are typically used for the catalysis of olefin metathesis [14], lignocellulosic biomass pyrolysis [15], or oxidative desulfurization of diesel fuels [16], and tungsten oxides are used as catalysts for propane dehydrogenation [17] or cellulose processing [18]. Molybdenum and tungsten oxides are Raman-active and their analysis using Raman spectroscopy has been described by Dieterle et al. [19] and Dieterle and Mestl [20] and Danile et al. [21], respectively. Therefore, these oxides were selected to analyze the usability of Raman spectroscopy for quantitative purposes during the quality control step in the production of catalysts.

In this study, we aimed to create models for determining the Mo contents of Mo-supported SBA-15 (Mo/SBA-15) and Mo-supported BEA (Mo/BEA) catalysts using calibration series. The chemometric parameters of these models were compared to determine the influence of the carrier on the development and optimization of the models. Furthermore, a model for determining the W content of the W-supported SBA-15 (W/SBA-15) catalysts was developed and optimized, as an example of the usefulness of Raman spectroscopy for the quantitative evaluation of other metals. In the last part of the study, a temperature control stage was paired with a Raman dispersion microscope to study the evolution of the Mo/BEA, Mo/SBA-15, and W/SBA-15 catalysts during calcination, and the results were used to optimize and elucidate the mechanism of this common catalyst treatment. We anticipate that this fast and efficient technique can be used to monitor the production process of catalysts.

## 2. Results and Discussion

### 2.1. Mo/SBA-15 Catalysts

The Mo/SBA-15 catalysts were synthesized via the impregnation of ammonia heptamolybdate (AHM) on SBA-15 followed by calcination, when the O=Mo=O bonds were formed.

**Figure 1:**
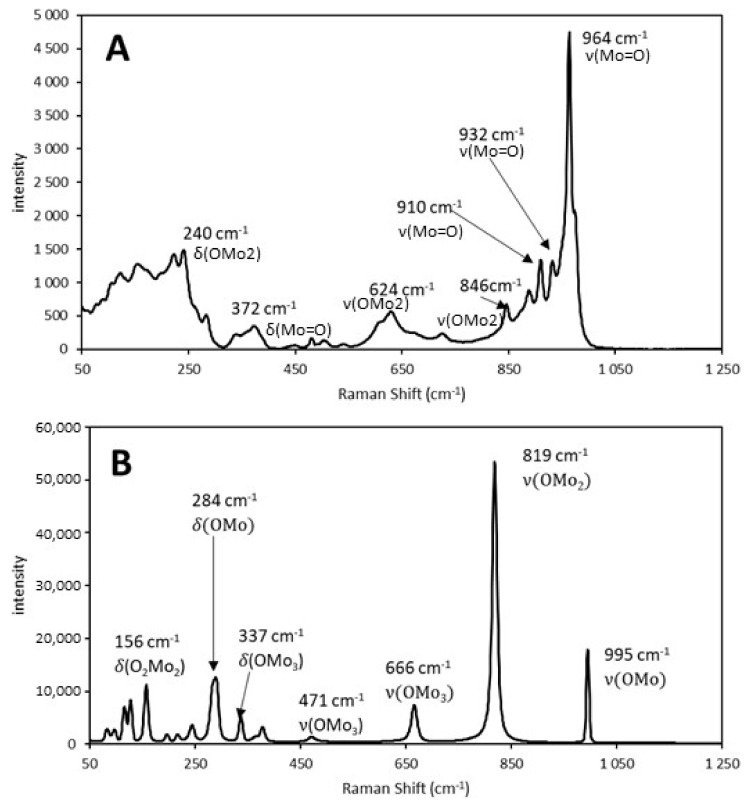
Raman spectra of the Mo-supported mesoporous silica catalyst with a Mo content of 38 wt% (**A**) before calcination (ammonium heptamolybdate) and (**B**) after calcination at 500 °C for 4 h (molybdenum (VI) oxide). A 532 nm laser was used to excite the samples. Here, δ and ν denote bending and stretching vibrations, respectively [22,23].

The Raman spectra of the 38% Mo/SBA-15 catalyst before and after calcination are illustrated in Figure 1.

After AHM was impregnated in the support, its characteristic Raman vibration bands at 964 and 932 cm^−1^ could be observed (Figure 1A). At this stage, the bonds between Mo, which is the main component of the catalyst, and the support, which did not participate in catalysis but increased the surface area for catalytic reactions, were weak. During calcination, AHM decomposed to molybdenum (VI) oxide (MoO_3_) and stronger bonds were formed between the Mo atoms and support. The characteristic vibration bands of MoO_3_ at 995 and 819 cm^−1^ could be observed in the Raman spectrum of the calcined catalyst (Figure 1B).

Because the bonds between Mo and the solid support are important for catalytic reactions, the Raman spectra of the calcined catalyst samples were used to quantify the Mo content of the catalysts.

**Figure 2:**
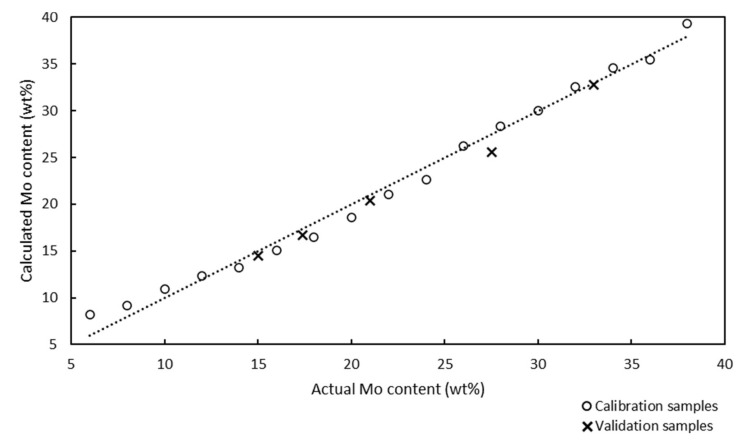
Raman model for the quantitative analysis of Mo-supported mesoporous silica catalysts with Mo contents in the range of 6–38 wt%. Correlation coefficient: 0.9943, one factor used. The circles and × marks represent the calcinated calibration and validation samples, respectively. Electronic supplementary material, Table S2.

The Raman spectra of 17 calibration samples were used to develop a Raman model for the quantitative analysis of the Mo content of the catalysts. Chemometric calculations in partial least squares (PLS) regression were performed in the spectral region between 61 and 1160 cm^−1^, and the Raman spectra were not preprocessed. The Raman model for the quantitative analysis of the Mo/SBA-15 catalysts is presented in Figure 2.

The x and y axes in the Raman model represent the actual and calculated Mo contents of the calcinated catalyst samples, respectively.

The Raman model was developed using 17 calibration samples with Mo contents in the range of 6–38 wt%, and was described using the correlation coefficient and chemometric errors, such as the root mean square error of calibration (RMSEC), root mean square error of prediction (RMSEP), and root mean square error of cross-validation (RMSECV). The ideal model should contain equally distributed calibration samples over the entire analysis range and its slope should be as close to 1.0000 as possible. The correlation coefficient of the model was determined to be 0.9943, and this value, which was close to 1.0000, confirmed the good correlation between the Raman spectra and the Mo contents of the samples. The RMSEC value can be calculated according to Equation (1):

(1) $$\mathrm{RMSEC}=\sqrt{\frac{\sum {\left(A_{ic}-A_{ia}\right)}^{2}}{m_{A}}}$$

where *A_ic_* and *A_ia_* are the calculated and actual Mo contents of the i-th calibration sample, respectively, and *m_A_* is a number of the calibration samples.

The RMSEC value of the Raman model developed for the quantitative analysis of Mo was determined to be 1.04%. The low RMSEC value indicated that the model was accurate; however, this parameter is an optimistic estimation of the model’s performance.

In addition to chemometric diagnostics, cross-validation can be used for the internal validation of models. Cross-validation involves separating each calibration sample from the model and using it as a validation sample. The cross-validation model is compiled using the results of the internal validation testing, and the result of this diagnostic is the RMSECV value. RMSECV provides more realistic determination errors than RMSEC, and the RMSECV value should not be significantly (several times) different than the RMSEC value. The RMSECV value of the Raman model used for the quantitative analysis of Mo was determined to be 1.20%, which was close to the RMSEC value; therefore, the Raman model should be accurate.

The RMSEP value describes the predictive ability of a calibration model. This value is obtained using internal validation, particularly when not enough validation samples are available, and can be calculated according to Equation (2):

(2) $$\mathrm{RMSEP}=\sqrt{\frac{\sum {\left(C_{ic}-C_{iv}\right)}^{2}}{m_{V}}}$$

where *C_ic_* and *C_iv_* are the calculated and actual Mo contents of the i-th validation sample, respectively, and *m_V_* is the number of validation samples.

The lower the RMSEP value, the higher the predictive ability of the model. The RMSEP value of the Raman model was determined to be 0.48%, which indicated the good predictability of the model.

**Figure 3:**
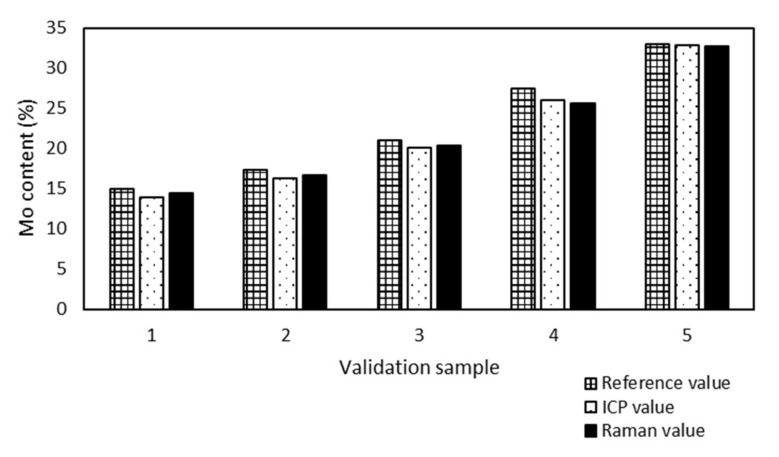
Comparison of inductively coupled plasma optical emission spectroscopy (ICP-OES) and Raman values of Mo content of validation samples supported SBA-15. The maximum absolute difference is 0.6% (sample 1) and the minimum is 0.1% (sample 5).

All validation samples were measured by ICP-OES because of the metal content and the results were compared with the Raman values (Figure 3) to prove the accuracy of the model prediction.

Samples no. 1 and 5 presented the maximum (0.6%) and minimum (0.1%) absolute differences between the Mo content determined by ICP-OES and Raman spectroscopy. The Raman spectroscopy results were in good agreement with the ICP-OES results, although the model was developed from sample preparation reference values.

Models developed using PLS regression employ a number of factors for chemometric calculations and prediction. Each factor is an independent source of variability for the calibration data. The factors are sorted according to the amounts of variability they represent. The first factor describes the most variability of the calibration samples and each additional factor describes most of the remaining variability. However, the first factor accounts for most of the common information contained in the data. The remaining factors describe more specific information, which represent small changes in the data, but are often important for analysis. When a low number of calibration factors are used, the probability of false positives is low because fewer variables are included in the calculations. The Raman model for the quantitative analysis of Mo used one factor, which minimized the probability of false positive results.

**Figure 4:**
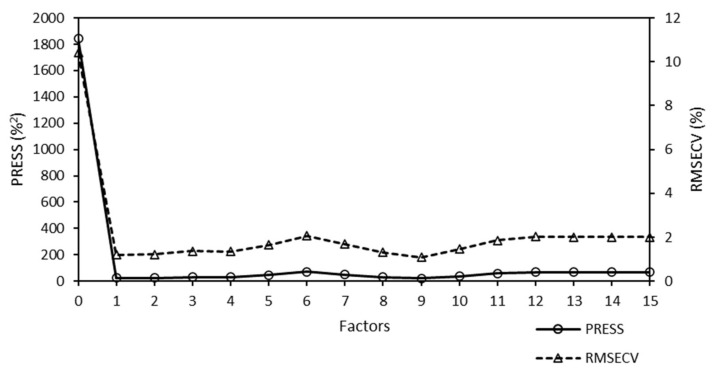
Predicted residual error sum of squares (PRESS) diagnostic function of the Raman model used to analyze the Mo content of Mo-supported mesoporous silica catalysts. The minimum PRESS/RMSECV value was achieved using one factor. Here, RMSECV denotes the root mean square error of cross-validation.

A predicted residual error sum of squares (PRESS) diagnostic function was used to develop and optimize the model. This diagnostic function revealed that the PRESS changed with the number of factors used to calibrate the components of the active PLS regression. The PRESS function was another indicator of the PLS regression calibration error. After a factor that represented the applicable information was added to the calibration model, the error of the model was reduced. At some point, the PRESS function reached a minimum and remained constant. If factors were added after this point, the performance of the method would not improve, but could decrease, owing to overfitting. The PRESS diagnostic function of the Raman model is presented in Figure 4.

The PRESS diagnostic curve presented a steep decreasing slope. The minimum of the PRESS function was achieved using one factor. The error for the model with one factor remained almost constant, and an increase in the number of factors did not bring any significant information.

All chemometric parameters of the model (correlation coefficient, RMSEC, RMSEP, RMSECV, and PRESS) and ICP-OES results confirmed that the model was suitable for the quantitative determination of the Mo content of Mo/SBA-15 catalysts.

### 2.2. W/SBA-15 Catalysts

Because we confirmed that Raman spectroscopy was suitable for the quantitative analysis of Mo, we subsequently tested W/SBA-15 catalysts using the same method. The characteristic bands of tungstate (975 and 887 cm^−1^) were observed in the Raman spectrum of the sample obtained after the impregnation of AMT on SBA-15.

**Figure 5:**
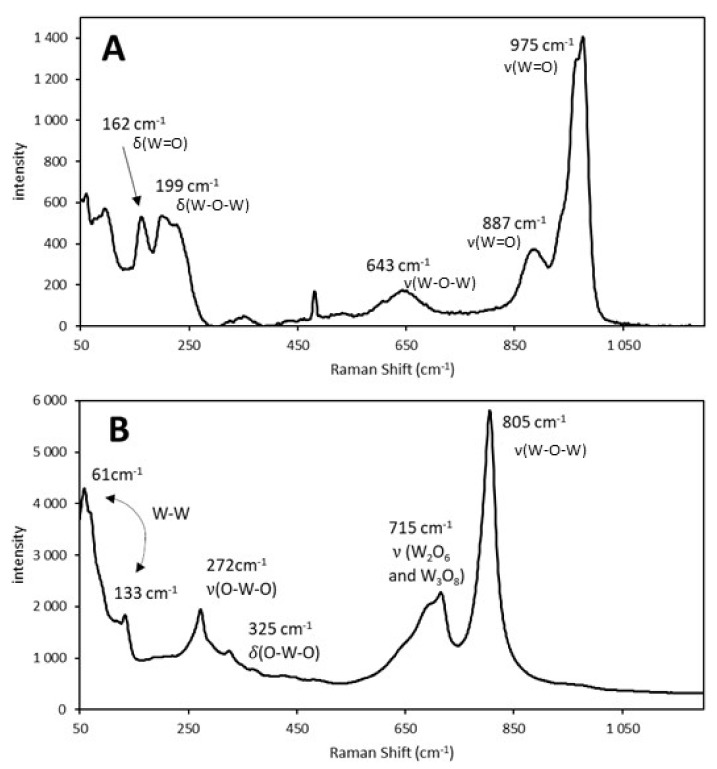
Raman spectra of the W-supported mesoporous silica catalyst with a W content of 40 wt% (**A**) before calcination (ammonium metatungstate) and (**B**) after calcination at 500 °C for 4 h (tungsten oxide). A 532 nm laser was used to excite the samples. Here, δ and ν denote bending and stretching vibrations, respectively [24,25].

The Raman spectra of the 40% W/SBA-15 catalyst before and after calcination are illustrated in Figure 5A,B, respectively.

During calcination, AMT decomposed to tungsten oxides (Figure 5B) and ammonia and water were simultaneously produced. Calcination led to the formation of specific bonds between W and the catalyst support, which were not present in the uncalcined catalyst sample. The support of the calcined catalyst, which contained tungsten oxides as active components, provided a large surface area for catalytic reactions. Calcined W/SBA-15 catalyst samples were used to develop a calibration model for the quantitative determination of their W content, using the WO_3_ vibration bands in their Raman spectra. Furthermore, chemometric calculations in PLS regression were performed in the spectral region between 50 and 1197 cm^−1^.

The Raman spectra used for the development and optimization of the Raman model did not require preprocessing (smoothing, derivative, filters) because the chemometric parameters of the model were suitable.

**Figure 6:**
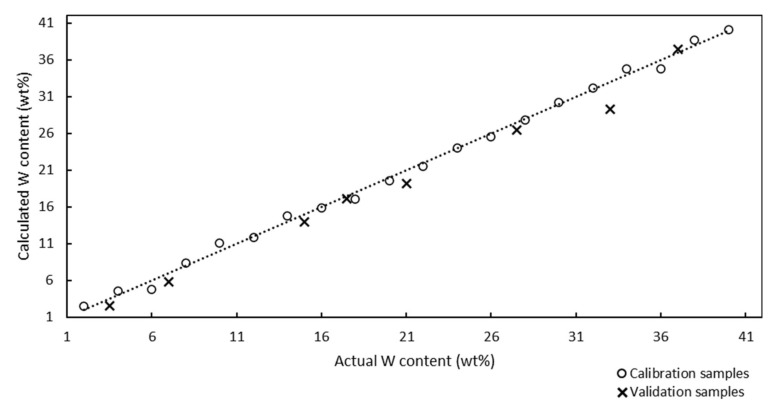
Raman model for the quantitative analysis of W-supported mesoporous silica catalysts with W contents in the range of 2–40 wt%. Correlation coefficient: 0.9984, four factors used. The circles and × marks represent the calcinated calibration and validation samples, respectively. Electronic supplementary material, Table S1.

The Raman model for the quantitative analysis of W in W/SBA-15 catalysts is illustrated in Figure 6.

The Raman model was developed using 20 calibration samples, and 8 samples were used for validation. The correlation coefficient of the model was determined to be 0.9984, which demonstrated the good correlation between the Raman spectra and W contents of the catalysts. The RMSEC value calculated using Equation (1) was determined to be 0.65%.

Internal validation (cross-validation diagnostic) was used to determine the predictability of the model. The RMSECV value of the cross-validation diagnostic testing was determined to be 1.29%. The RMSECV value represents a more realistic error of the determination than the RMSEC value; nevertheless, the small difference (0.64%) between these types of errors confirmed the accuracy of the Raman model for the determination of the W content of W/SAB-15 catalysts and the minimal probability of false positive results. The RMSEP value calculated using Equation (2) was 0.70%. This value was intermediate between the RMSEC and RMSECV values. The RMSEP value was more realistic than the RMSEC and less than the RMSECV values, respectively. According to the chemometric parameters (RMSEC, RMSEP, and RMSECV), the maximum error of the Raman model should be 1.29%.

**Figure 7:**
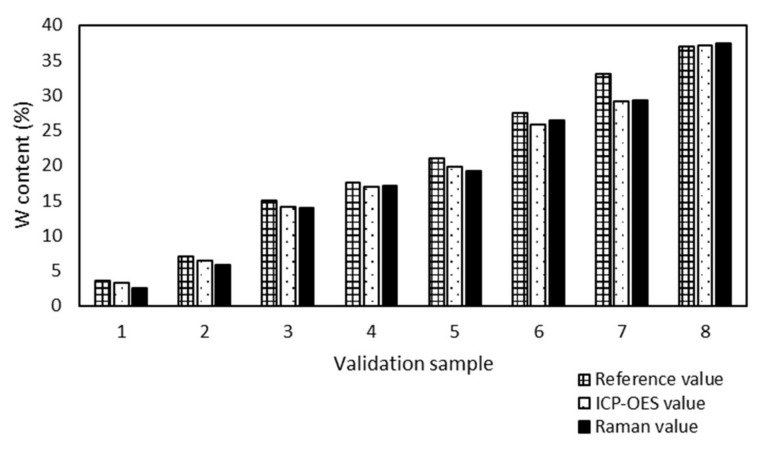
Comparison of ICP-OES and Raman values of W content of validation samples supported SBA-15. The maximum absolute difference is 0.7% (samples 1, 2, and 6) and the minimum is 0.2% (samples 3, 4, and 7).

The analytical method ICP-OES was used for accuracy determination of the prediction of the Raman model. The results, determined by ICP-OES and predicted by the Raman model, were compared (Figure 7).

All ICP-OES values of W/SBA-15 validation samples had the absolute difference under 1% from the Raman values. The maximum absolute difference 0.7% was found out for 3 samples (sample 1, 2 and 6) and the minimum difference 0.2% also for 3 samples (sample 3, 4 and 7). The Raman spectroscopy proved to be a suitable tool for the determination of the W content in W/SBA-15 samples.

**Figure 8:**
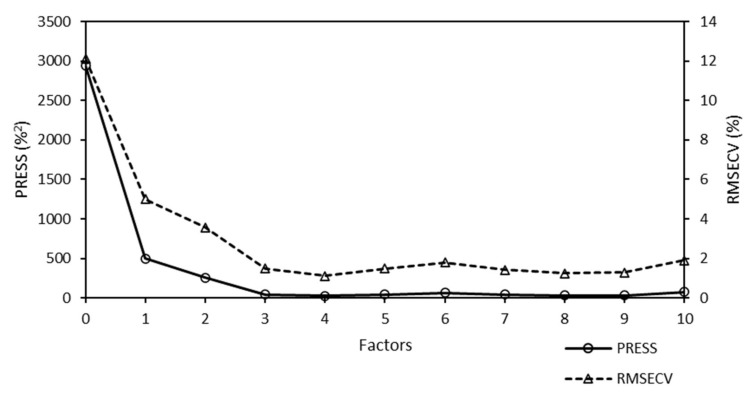
Predicted residual error sum of squares (PRESS) diagnostic function of the Raman model used for the analysis of the W content of W-supported mesoporous silica catalysts. The minimum PRESS/RMSECV value was achieved using four factors. Here, RMSECV denotes the root mean square error of cross-validation.

The PRESS diagnostic curve of the Raman model for the quantitative analysis of W is presented in Figure 8.

The sharp decrease in the PRESS function was fulfilled for the Raman model. The minimum PRESS/RMSECV value was achieved using four factors for chemometric calculations. More factors were required for the Raman model used for the quantitative analysis of W than for the Raman model used for the quantitative analysis of Mo (one factor). However, it was not such a number that would lead to a re-fitting of the model and would introduce a higher probability of incorrect prediction. The increase in the number of factors did not significantly affect the predictability of the model.

### 2.3. Mo/BEA Catalysts

The Raman model for the quantitative analysis of the Mo/BEA was also developed and determined the effect of the support on the accuracy of the model. Because MoO_3_ is Raman-active, and we already demonstrated that the proposed Raman model could be used for its quantitative determination (Section 2.1.), we proceeded to analyze Mo/BEA, another Mo-supported catalyst. BEA is a common support for catalysts; moreover, BEA is Raman-inactive, and therefore, it should not interfere with the Raman spectra of the Mo/BEA catalysts.

**Figure 9:**
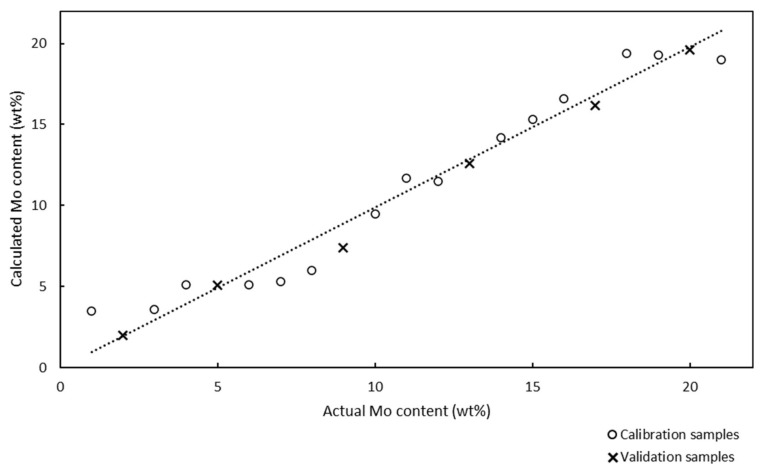
Raman model for the quantitative analysis of Mo-supported beta zeolite catalysts with Mo contents in the range of 1–21 wt%. Correlation coefficient: 0.9777, two factors used. The circles and × marks represent the calcinated calibration and validation samples, respectively. Electronic supplementary material, Table S3.

The Raman spectra of 15 Mo/BEA calibration samples with Mo contents in the range of 1–21 wt% were obtained. We could not prepare calibration samples with higher Mo contents because of the low absorbability of BEA for AHM. The Raman model for quantitative analysis of Mo/BEA is presented in Figure 9.

Chemometric calculations in PLS regression were performed in the spectral region between 73 and 1161 cm^−1^. The Raman spectra did not require preprocessing because the chemometric parameters (correlation coefficient, RMSEC, RMSEP, and RMSECV) were suitable. The correlation coefficient was 0.9777, which indicated a satisfactory correlation between the Raman spectra and amount of Mo in the Mo/BEA catalyst samples. However, this correlation coefficient was lower than that for the Mo/SBA-15 catalyst samples, which was probably due to the different support. Internal validation was performed using six validation samples.

The RMSEC, RMSECV, and RMSEP values were 1.25%, 1.78%, and 0.90%, respectively. These chemometric parameters were higher than those of the Raman model used to analyze Mo/SBA-15, and this was ascribed to the different supports and correlation coefficients for the two types of catalysts. However, these parameters still demonstrated that the proposed Raman model was suitable for the quantitative determination of Mo in Mo/BEA catalysts. However, the model might not predict the Mo content of Mo/BEA with the same accuracy that it predicted the Mo content of Mo/SBA-15 (lower correlation coefficient, higher RMSEC, RMSECV, and RMSEP compared to Mo/SBA-15).

**Figure 10:**
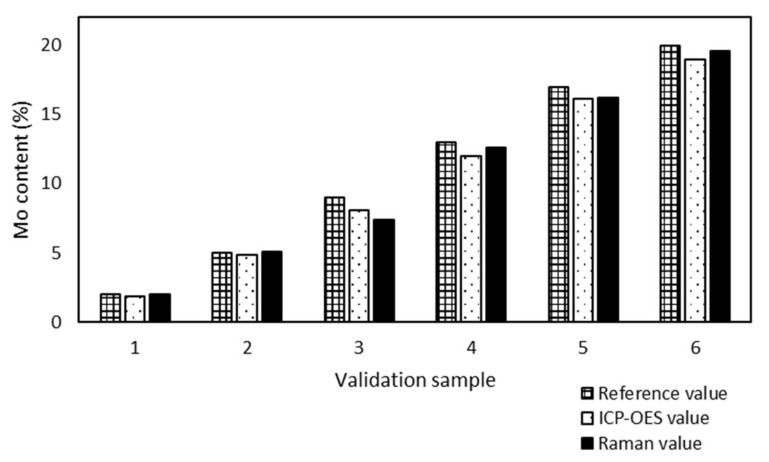
Comparison of ICP-OES and Raman values of Mo content of validation samples supported BEA. The maximum absolute difference is 0.7% (sample 3) and the minimum is 0.1% (sample 1 and 5).

The accuracy of the Raman model was proved by the results of six validation samples from ICP-OES (Figure 10).

The maximum absolute difference between ICP-OES and Raman values of validation samples of Mo/BEA was found to be 0.7% (sample 3) and the minimum 0.1% for samples 1 and 5. As in the case of Mo/SBA-15 and W/SBA-15, ICP-OES also confirmed the accuracy of the prediction of the developed Raman model from the sample preparation reference values. The difference in the determination was acceptable for these analytical methods.

**Figure 11:**
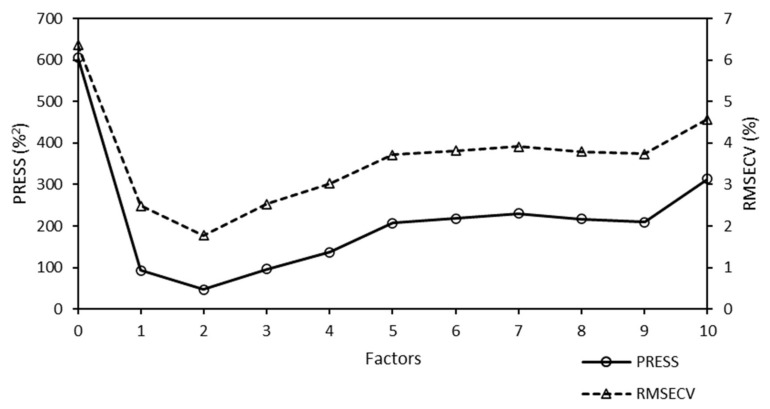
Predicted residual error sum of squares (PRESS) diagnostic function of the Raman model used for the analysis of the Mo content of Mo-supported beta zeolite catalysts. The minimum PRESS/RMSECV value was achieved using two factors. Here, RMSECV denotes the root mean square error of cross validation.

The diagnostic PRESS curve of the Raman model for Mo/BEA is illustrated in Figure 11.

The minimum diagnostic PRESS function was achieved in PLS regression using two factors for chemometric calculations. The shape of the PRESS curve for this model was not optimal (Figure 11). RMSECV increased because two factors were used for analysis, and also because the support was changed (BEA was used instead of SBA-15).

These results demonstrated that Raman spectroscopy was a suitable method for the determination of the Mo content of Mo/BEA catalysts; nevertheless, the support type affected the development and optimization of the Raman model for the quantitative analysis of Mo. Therefore, the developed model was not universal, and new Raman models should be developed and optimized depending on the supports used.

### 2.4. Analysis of the Calcination of Catalysts Using Raman Spectroscopy with Temperature Control Stage

A DXR (Thermo Fisher Scientific) Raman microscope equipped with a THMS600 (Linkam Scientific Instruments) heating and freezing stage system was used to study the calcination of the Mo/SBA-15, W/SBA-15, and Mo/BEA catalysts. The calcination of the Mo- and W-supported catalysts led to the formation of MoO_3_ from AHM and WO_3_ from AMT, respectively.

A low temperature ramp (1 °C/min) was selected for the calcination to facilitate the formation of strong bonds between metals and supports. A higher temperature ramp could lead to the decomposition of the deposited compounds (molybdate, tungstate), which would prevent the formation of active metal oxide species. We selected catalysts with high metal contents (24% Mo/SBA-15, 38% W/SBA-15, and 21% Mo/BEA) for these experiments, and the W content was higher than those of Mo because the intensity of the WO_3_ Raman peaks is lower than that of MoO_3_.

**Figure 12:**
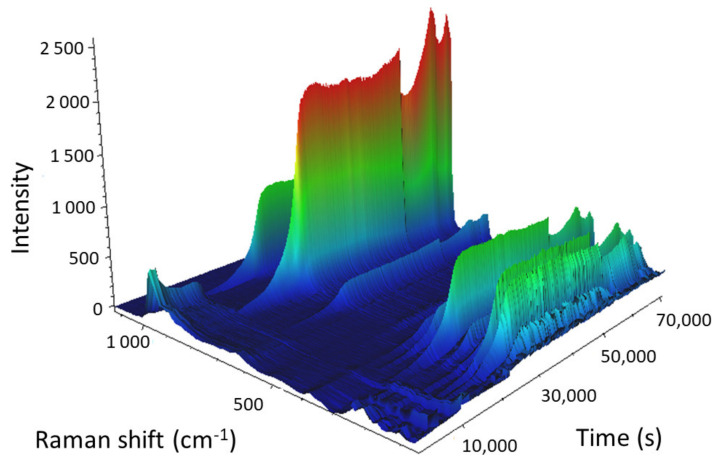
3D view of the calcination of the Mo-supported mesoporous silica catalyst with a Mo content of 24 wt%. Heating: starting temperature 25 °C; temperature gradient 1 °C/min; end temperature 500 °C (in 28,738 s). Isotherm for 4 h (43,072 s). Cooling: start temperature 500 °C; temperature ramp 1 °C/min; end temperature 50 °C (70,383 s).

The 3D view of the calcination of the 24% Mo/SBA-15 catalyst is presented in Figure 12.

The calcination of AHM can be described as follows:

(3) $${\left(NH_{4}\right)}_{6}Mo_{7}O_{24}\;\cdot 4H_{2}O\;\to {\left(NH_{4}\right)}_{6}Mo_{7}O_{24}+4H_{2}O\;\to 7MoO_{3}+\;3H_{2}O+6NH_{3}$$

Because the bound hydrates were not of interest, we focused on the spectral region that contained the vibration bands of AHM, which were subsequently transformed into the vibration bands of MoO_3_. At the beginning of the calcination of Mo/SBA-15 (25 °C), the main vibration band of AHM (964 cm^−1^) was observed in the Raman spectrum; however, as the temperature increased, the Raman intensity of the main band decreased and the band completely disappeared when the temperature reached 220 °C (11,580 s into the experiment). When AHM was decomposed and water and ammonia were released, the characteristic vibration bands of AHM disappeared. As the temperature was further increased (above 220 °C), O–Mo bonds started to form, and the intensities of the MoO_3_ vibration bands (particularly the bands at 995 and 819 cm^−1^) gradually increased. All MoO_3_ vibration bands reached maximum intensity 6.7 min after isothermal mode at 500 °C was reached. The sharp decrease in the intensities of the Raman bands (55,488 s; cooling at 295.3 °C) was caused by a change in objective focus during measurements.

These data indicated that the duration of the calcination process could be shortened by approximately half, which would save time and energy.

**Figure 13:**
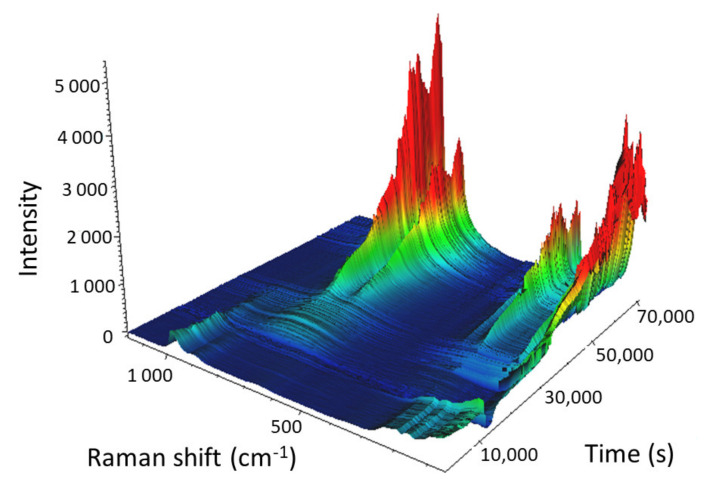
3D view of the calcination of the W-supported mesoporous silica catalyst with a W content of 38 wt%. Heating: start temperature 25 °C; temperature gradient 1 °C/min; end temperature 500 °C (in 28,738 s). Isotherm for 4 h (43,072 s). Cooling: start temperature 500 °C; temperature ramp 1 °C/min; end temperature 50 °C (70,383 s).

The 3D view of the calcination of the 38% W/SBA-15 catalyst is illustrated in Figure 13.

The calcination of AMT can be described as follows:

(4) $${\left(NH_{4}\right)}_{6}H_{2}W_{12}O_{40}\;\cdot \;\times H_{2}O\;\to {\left(NH_{4}\right)}_{6}H_{2}W_{12}O_{40}+\times H_{2}O\;\to 12WO_{3}+\;4H_{2}O+6NH_{3}$$

The bound hydrates were not of the interest; therefore, for these measurements, we focused on the spectral region that contained the characteristic bands of AMT.

The main Raman vibration bands of AMT (975 and 887 cm^−1^) were observed at the beginning of the calcination process. As the temperature increased, their intensities decreased, and the bands disappeared from the spectrum at 257 °C (13,962 s) when AMT decomposed and formed water and ammonia. The intensities of the 805 and 715 cm^−1^ Raman vibration bands started to increase 31 min into the experiment in isothermal mode at 500 °C. The intensities of these bands continued to increase during the subsequent 3.5 h, and also, when the sample was cooled from 500 to 66 °C (which led to the complete formation of WO_3_). The sharp decrease in the intensity of the Raman spectrum (66,506 s; cooling at 110.8 °C) was caused by a change in objective focus during the measurement, similar to that reported for Mo/SBA-15.

These results indicated that the calcination of the W/SBA-15 catalyst could not be accelerated to save time and energy.

**Figure 14:**
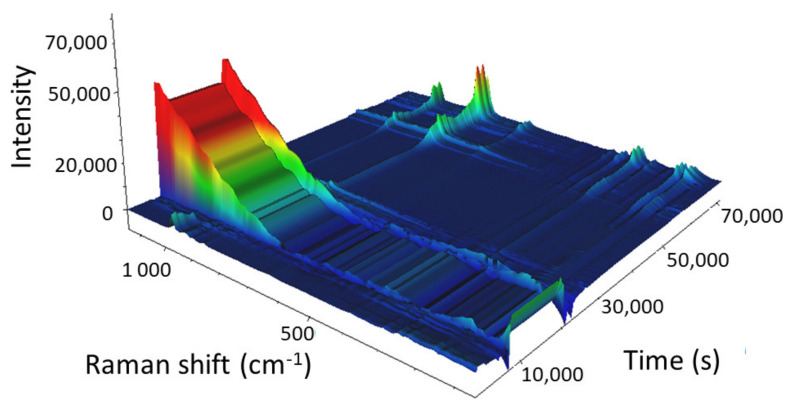
3D view of the calcination of the Mo-supported beta zeolite catalyst with a Mo content of 21 wt%. Heating: start temperature 25 °C; temperature gradient 1 °C/min; end temperature 500 °C (in 28,738 s). Isotherm for 4 h (43,072 s). Cooling: start temperature 500 °C; temperature ramp 1 °C/min; end temperature 50 °C (70,383 s).

The 3D view of calcination of the 21% Mo/BEA catalyst is presented in Figure 14.

The Raman vibration bands of AHM were observed from the beginning of the experiment until the calcination temperature reached 109 °C (4989 s). After the catalyst structure disintegrated, the fluorescence of the BEA support increased until the temperature reached 420 °C (23,859 s). Owing to the significant fluorescence of BEA, it was not possible to observe the changes in the vibration bands of AHM to MoO_3_. When the temperature reached 420 °C, the fluorescence of BEA disappeared, and the increase in the intensity of the MoO_3_-specific bands (995 and 819 cm^−1^) was observed until the temperature reached 62 °C during cooling. Therefore, it was not possible to shorten the duration of the calcination of Mo/BEA to save energy and time. The differences observed during the calcination of the Mo-supported catalysts were attributed to the absorbency of BEA being lower than that of SBA-15, which indicated that the process required to anchor Mo to BEA should be longer than that required to anchor Mo to SBA-15.

Raman spectroscopy combined with the temperature control stage is an interesting analytical tool that can facilitate the study of structural changes in catalysts during processes that involve heating or occur isothermally. Based on the obtained data (temperature, time), we concluded that it would be possible to optimize the production of catalysts and minimize operating costs. Our results demonstrated that calcination affected not only the metal but also the catalyst support; therefore, to optimize processing, each catalyst should be approached individually.

## 3. Materials and Methods

### 3.1. Sample Preparation

The powdered catalyst supports—SBA-15 (UniCRE a.s.) and BEA CP814E (Zeolist, Si/Al = 12.5)—were calcined at 540 °C for 6 h (temperature ramp of 1 °C/min) followed by impregnation via the incipient wetness impregnation method using aqueous solutions of ammonium heptamolybdate ((NH_4_)_6_Mo_7_O_24_·4H_2_O; AHM, p.a. purity, Lach-Ner s.r.o., Neratovice, Czech Republic) or ammonium metatungstate ((NH_4_)_6_H_2_W_12_O_40_·×H_2_O; AMT, ≥ 85 wt% WO_3_, Sigma-Aldrich, St. Louis, MO, USA) to obtain catalysts with Mo or W contents in the range of 1–40 wt%, respectively (Supplementary data). After the samples were dried at 120 °C for 12 h, they were homogenized using an agate mortar and pestle before calcination at 500 °C for 4 h at a temperature ramp of 1 °C/min.

Because the selected catalyst supports had no visible vibration bands using an excitation laser at 532 nm, no interferences in the Raman spectra of the oxidized metal species were expected.

### 3.2. Raman Spectroscopy

A DXR (Thermo Fischer Scientific, Waltham, MA, USA) Raman microscope was used to analyze the solid samples. Immediately after calcination, the samples were placed in nuclear magnetic resonance cuvettes and were analyzed using a 4× magnification lens (objective). A 532 nm laser with an exposure time of 1 s and a power of 10 mW (aperture slit of 10 µm) was used for sample excitation. The obtained Raman spectra were by the means of 500 scans. All spectra were processed using the TQ Analyst 9 (Thermo Fischer Scientific) software in partial least square (PLS) regression and were used to develop and optimize the Raman quantitative models for Mo and W. No preprocessing techniques (smoothing, derivatives) were used for the Raman spectra, and the spectral range was selected individually for each quantitative model.

### 3.3. Raman Spectroscopy with Temperature Control Stage

The catalyst calcination processes were studied using a DXR (Thermo Fisher Scientific) Raman microscope equipped with a THMS600 (Linkam Scientific Instruments, Tadworth, Surrey, United Kingdom) heating and freezing stage system. The 21% Mo/BEA, 24% Mo/SBA-15, and 38% W/SBA-15 uncalcined samples (approximately 20 mg) were added to quartz crucibles, were placed on the stage, and were observed with a 50× magnification lens. The aperture, exposure time, wavelength, and laser excitation power were the same as those used for the Raman spectroscopy measurements. The samples were heated at a temperature ramp of 1 °C/min from 25 to 500 °C and were maintained at 500 °C for 4 h. Subsequently, the samples were cooled at a temperature ramp of 1 °C/min. The data were processed using the OMNIC 9 (Thermo Fisher Scientific) software.

## 4. Conclusions

In this study, we demonstrated that Raman spectroscopy can be a suitable analytical method for the quantitative analysis of Mo/SBA-15, W/SBA-15, and Mo/BEA catalysts. The developed and optimized calibration models were characterized using chemometric parameters, such as RMSEC, RMSECV, RMSEP, the correlation coefficient, and PRESS diagnostic. The values of these parameters demonstrated that the models were adequate for determining the Mo and W contents of the analyzed catalysts. Moreover, we demonstrated that changing the catalyst support affected the model parameters and its predictability. Therefore, when changing the catalyst support, it is necessary to develop a new fully functional model for the quantitative determination of the metal content of catalysts. Lastly, we combined Raman spectroscopy with temperature control stage to study the calcination of the Mo/BEA, Mo/SBA-15, and W/SBA-15 catalysts. The results indicated that each catalyst should be analyzed individually during processing, as calcination affected not only the metal, but also the support.

We believe that our results (calibration models, calcination analysis data) should be particularly beneficial for molybdenum and tungsten catalyst manufacturing.

## Supplementary Materials

The following are available online: Table S1: Calibration and validation samples for the Raman model for the W-supported on mesoporous silica catalysts, Table S2: Calibration and validation samples for the Raman model for the Mo-supported on mesoporous silica catalysts, Table S3: Calibration and validation samples for the Raman model for the Mo-supported on beta zeolite catalysts.
