# Supplementary material for: Raman Spectroscopy as Molybdenum and Tungsten Content Analysis Tool for Mesoporous Silica and Beta Zeolite Catalysts
Source: Molecules. 2020 Oct 23;25(21):4918. doi: 10.3390/molecules25214918 (PMC7660599; doi:10.3390/molecules25214918)
Supplement: Supplementary file 1 [file molecules-25-04918-s001.pdf]

molecules

Supplementary Material

Raman Spectroscopy as Molybdenum and Tungsten Content Analysis Tool for Mesoporous Silica and Beta Zeolite Catalysts

Romana Velvarská *, Zdeněk Tišler, Veronika Raichlová and José Miguel Hidalgo-Herrador

Unipetrol Centre for Research and Education, Revoluční 1521/84, 400 01 Ústí nad Labem, Czech Republic; zdenek.tisler@unicre.cz (Z.T.); veronika.raichlova@unicre.cz (V.R.); jose.hidalgo@unicre.cz (J.M.H.-H.)

* Correspondence: romana.velvarska@unicre.cz

Table S1. Calibration and validation samples for the Raman model for the W-supported on mesoporous silica catalysts.

Calibration Sample No.	Actual Content (wt%)	Calculated Content (wt%)
1	2	2.5
2	4	4.5
3	6	4.8
4	8	8.3
5	10	11.1
6	12	11.8
7	14	14.8
8	16	15.9
9	18	17
10	20	19.5
11	22	21.5
12	24	24
13	26	25.5
14	28	27.8
15	30	30.2
16	32	32.2
17	34	34.8
18	36	34.8
19	38	38.7
20	40	40.1
**Validation Sample No.**	**Actual Content (wt%)**	**Calculated Content (wt%)**
1	3.5	2.5
2	7	5.8
3	15	13.9
4	17.5	17.1
5	21	19.2
6	27.5	26.5
7	33	29.3
8	37	37.4

Table S2. Calibration and validation samples for the Raman model for the Mo-supported on mesoporous silica catalysts.

Calibration Sample No.	Actual Content (wt%)	Calculated Content (wt%)
1	6	8.2
2	8	9.2
3	10	10.9
4	12	12.3
5	14	13.2
6	16	15.1
7	18	16.5
8	20	18.6
9	22	21
10	24	22.6
11	26	26.2
12	28	28.3
13	30	30
14	32	32.6
15	34	34.6
16	36	35.5
17	38	39.3
**Validation Sample No.**	**Actual Content (wt%)**	**Calculated Content (wt%)**
1	15	14.5
2	17.4	16.7
3	21	20.4
4	27.5	25.6
5	33	32.8

Table S3. Calibration and validation samples for the Raman model for the Mo-supported on beta zeolite catalysts.

Calibration Sample No.	Actual Content (wt%)	Calculated Content (wt%)
1	1	3.5
2	3	3.6
3	4	5.1
4	6	5.1
5	7	5.3
6	8	6
7	10	9.5
8	11	11.7
9	12	11.5
10	14	14.2
11	15	15.3
12	16	16.6
13	18	19.4
14	19	19.3
15	21	19
**Validation Sample No.**	**Actual Content (wt%)**	**Calculated Content (wt%)**
1	2	2
2	5	5.1
3	9	7.4
4	13	12.6
5	17	16.2
6	20	19.6
